# Visualization of Moiré Magnons in Monolayer Ferromagnet

**DOI:** 10.1021/acs.nanolett.3c00417

**Published:** 2023-04-11

**Authors:** Somesh Chandra Ganguli, Markus Aapro, Shawulienu Kezilebieke, Mohammad Amini, Jose L. Lado, Peter Liljeroth

**Affiliations:** † Department of Applied Physics, Aalto University, FI-00076 Aalto, Finland; ‡ Department of Physics, Department of Chemistry and Nanoscience Center, University of Jyväskylä, FI-40014 Jyväskylä, Finland

**Keywords:** magnon, two-dimensional ferromagnet, moiré modulation, monolayer chromium tribromide, scanning tunneling microscopy and spectroscopy

## Abstract

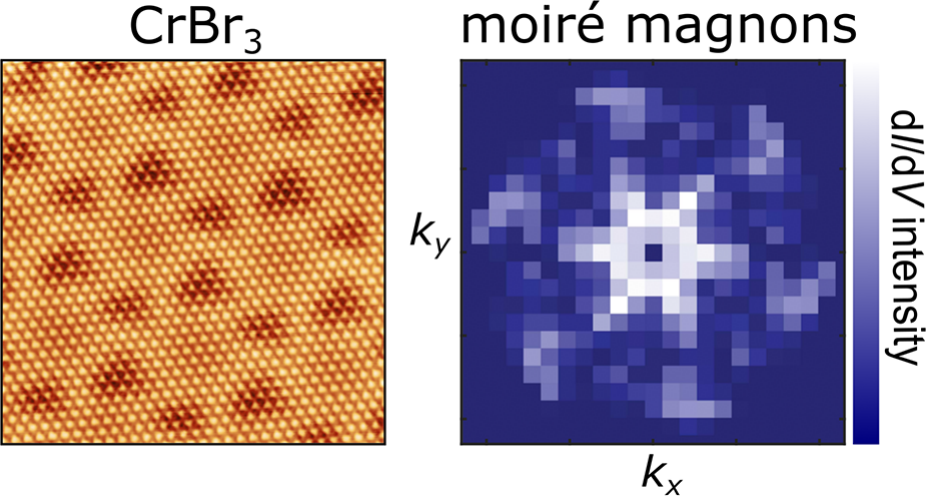

Two-dimensional magnetic materials provide an ideal platform to explore collective many-body excitations associated with spin fluctuations. In particular, it should be feasible to explore, manipulate, and ultimately design magnonic excitations in two-dimensional van der Waals magnets in a controllable way. Here we demonstrate the emergence of moiré magnon excitations, stemming from the interplay of spin-excitations in monolayer CrBr_3_ and the moiré pattern arising from the lattice mismatch with the underlying substrate. The existence of moiré magnons is further confirmed via inelastic quasiparticle interference, showing the appearance of a dispersion pattern correlated with the moiré length scale. Our results provide a direct visualization in real-space of the dispersion of moiré magnons, demonstrating the versatility of moiré patterns in creating emergent many-body excitations.

The recent discovery of two-dimensional van der Waals (vdW) monolayer magnetic materials has opened new avenues for scalable, defect-free samples for spintronic applications and artificial designer materials.^1−10^ It provides an exciting opportunity to control and manipulate magnetism in two-dimensions^11−14^ and create new emergent states in vdW heterostructures.^15−19^ A common feature of two-dimensional materials is the appearance of moiré patterns due to the lattice mismatch or twist between the monolayer and the substrate. Using the twist degree of freedom has emerged as a powerful strategy to design new quantum states.^20−23^ Paradigmatic examples are the emergent correlated and topological states in graphene moiré multilayers,^24,25^ ferroelectricity in hexagonal boron nitride moiré bilayers,^26^ moiré excitons in twisted MoSe_2_/WSe_2_,^27^ and moiré magnetism in CrI_3_ moiré bilayers.^28^ The emergence of moiré phenomena in magnetic van der Waals materials is a newly explored field, and in particular, the possibility of creating moiré magnon excitations remains an open problem in twistronics.

Chromium trihalides (CrX_3_, X = Cl, Br, and I, Figure 1a) have been established as a prominent family of 2D magnetic materials^29^ with all three showing ferromagnetic order, where the easy axis is out-of-plane for CrBr_3_^6,7^ and CrI_3_,^2^ and in-plane for CrCl_3_.^30^ We have carried out low-temperature scanning tunneling microscopy (STM) and spectroscopy (STS) to probe the magnon excitations in monolayer CrBr_3_. We show that the results can be understood in terms of moiré magnons arising from a reconstruction of the magnon dispersion by the moiré pattern formed by the lattice mismatch between CrBr_3_ and the substrate. This leads to new van Hove singularities in the magnon spectral function that are correlated with the moiré length scale. Furthermore, by employing quasiparticle interference with inelastic spectroscopy, we directly probe the magnon dispersion in reciprocal space, allowing us to map the moiré magnon spectra. Our results demonstrate the emergence of moiré magnons and the impact of moiré patterns on the magnetic excitations of 2D materials.

**Figure 1:**
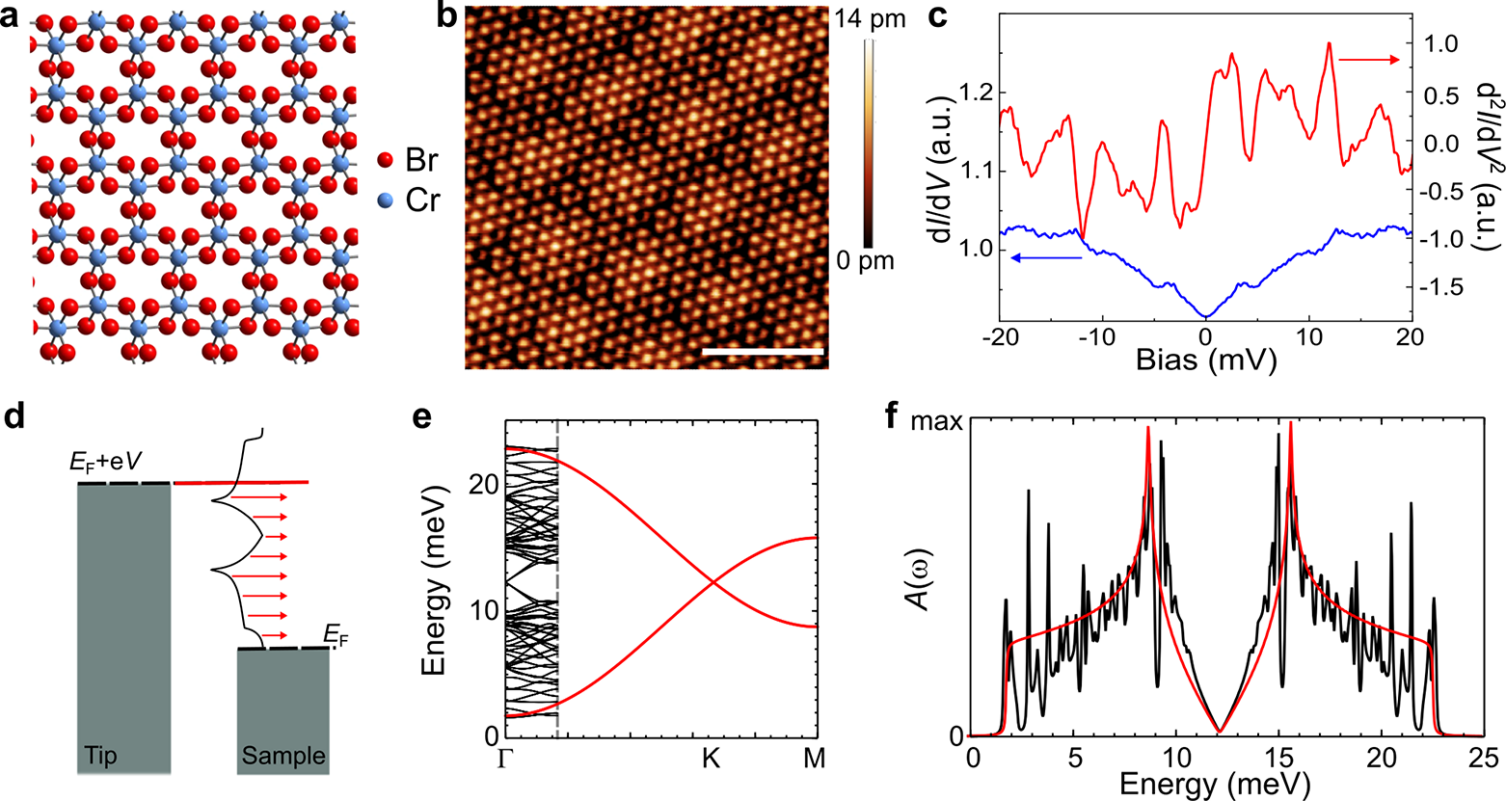
Probing moiré magnons in CrBr_3_ with inelastic tunneling spectroscopy. (a) Schematic of the CrBr_3_ structure. (b) Atomically resolved image of CrBr_3_ on HOPG. Image was taken at sample bias 1.5 V. Scale bar 5 nm. (c) Symmetrized d*I*/d*V* (blue) and numerically differentiated d^2^*I*/d*V*^2^ (red) obtained in monolayer CrBr_3_ on HOPG. (d) Schematic of inelastic tunneling spectroscopy of magnons. (e) Unfolded magnon bands and moiré-folded magnon mini-bands. (f) Unfolded (red) and moiré-folded (black) magnonic spectral functions.

We have carried out experiments on CrBr_3_ monolayers on a highly oriented pyrolytic graphite (HOPG) substrate at a *T* = 350 mK (see Supporting Information (SI) for more experimental details). Typical STM topography image (Figure 1b) shows both bright triangular protrusions arising from the bromine atoms in the CrBr_3_ layer as well as a longer length-scale variation corresponding to the moiré pattern, which arises from the lattice mismatch between the CrBr_3_ monolayer and the HOPG substrate. Magnetic excitations can be probed via inelastic tunneling spectroscopy (IETS) and they should result in bias-symmetric steps in the d*I*/d*V* signal.^6,31−33^ We observe clear inelastic excitations experimentally as demonstrated in Figure 1c that shows both the measured d*I*/d*V* (symmetrized) and numerically differentiated and smoothened d^2^*I*/d*V*^2^ signals (see SI for details). As schematically illustrated in Figure 1d, for a ferromagnetic system, we would expect the d*I*/d*V* to correspond to the integrated magnon density of states (DOS) while the d^2^*I*/d*V*^2^ signal directly corresponds to the local magnon spectral function. It is immediately obvious that our experimental d^2^*I*/d*V*^2^ contains many more peaks than expected for a typical magnon spectrum. We explain this discrepancy below as arising from the moiré-induced modification of the magnon spectrum.

The physics behind the moiré magnons can be understood starting from the anistropic Heisenberg Hamiltonian^34,35^ describing the spin excitations in a magnetic two-dimensional system (see SI for details)
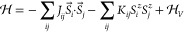
1with *J*_*ij*_ the spatially modulated isotropic exchange coupling, *K*_*ij*_ the anisotropic exchange, and *S*_*n*_^α^ the local *S* = 3/2 operators in the Cr atoms forming a honeycomb lattice (Figure 1a). The term  contains other potential terms in the Hamiltonian^4,34−36^ including Dzyaloshinskii–Moriya interaction, biquadratic exchange, single-ion anisotropy, and Kitaev interaction, which for the sake of simplicity are not included in the next discussion as their role is not important for the emergence of moiré magnons. The local moments at the Cr-sites have a ferromagnetic coupling via superexchange through Br atom, parametrized by *J*_*ij*_. The existence of the substrate leads to an additional exchange interaction mediated by the RKKY interaction. This substrate-mediated RKKY interaction depends on the local stacking between CrBr_3_ and HOPG, which in turn is controlled by the moiré modulation between HOPG and CrBr_3_. This modulation in real space leads to the change of the exchange constants *J*_*ij*_(7,28,37−39) and, in turn, the spin stiffness through the moiré unit cell.^40−43^ Moreover, potential small structural distortions lead to a modulation of the superexchange interaction, both of which follow the same periodicity as the moiré pattern. Holstein–Primakoff mapping^44^ allows the magnonic Hamiltonian to be written in terms of the bosonic magnon operators
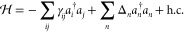
2with γ_*ij*_ ∼ *J*_*ij*_ controlling the spin stiffness and ⟨Δ_*n*_⟩ determines the magnon gap, and *a*_*n*_^†^, *a*_*n*_ are the creation and annhilation magnon operators. For CrBr_3_, first-principles calculations^45^ predict a bandwidth of the magnon spectra of ∼30 meV in the absence of a moiré pattern, and in the following we take that the moiré modulations changes the local exchange while keeping the global bandwidth approximately equal to the uniform case.

In the absence of the moiré pattern, the magnon dispersion features two magnon bands stemming from the two Cr atoms in the unit cell. The magnon dispersion shows Dirac points when neglecting small contributions coming from , and a low energy quadratic dispersion with a gap controlled by *K*. In the presence of the moiré pattern, the real-space modulation of γ_*ij*_ leads to the appearance of magnon mini-bands in the moiré supercell, as shown in Figure 1e. The multiple folding of the original moiré bands and induced anticrossings driven by the moiré exchange modulation gives rise to a whole new set of moiré singularities, as shown in Figure 1f.

We have carried out inelastic tunneling spectroscopy experiments (parameters mentioned in the SI) over a range of CrBr_3_ islands with different moiré periodicities allowing us to address the impact of the underlying moiré pattern on the magnetic excitations. Figure 2a,c shows the effect of the different moiré length scales (moiré wavelengths of 7 and 3.6 nm) on the inelastic excitations. The antisymmetrized d^2^*I*/d*V*^2^ (details in the SI) shows strong peaks that are spatially quite uniform as shown in Figure 2b,d. The moiré magnon features are expected to be the most visible at the bottom of the magnon band due to the folding into the moiré Brillouin zone (see Figure 1f). In addition, the higher energy inelastic excitations are less intense in the experimental spectra, which can be understood through magnon–magnon interation effects.^46^ Therefore, we focus on the lower energy features and comparing Figure 2b,d, it is clear that there are more inelastic features with a smaller energy spacing in the experiments on the larger length-scale moiré pattern (see SI for the statistics of inelastic peak energies).

**Figure 2:**
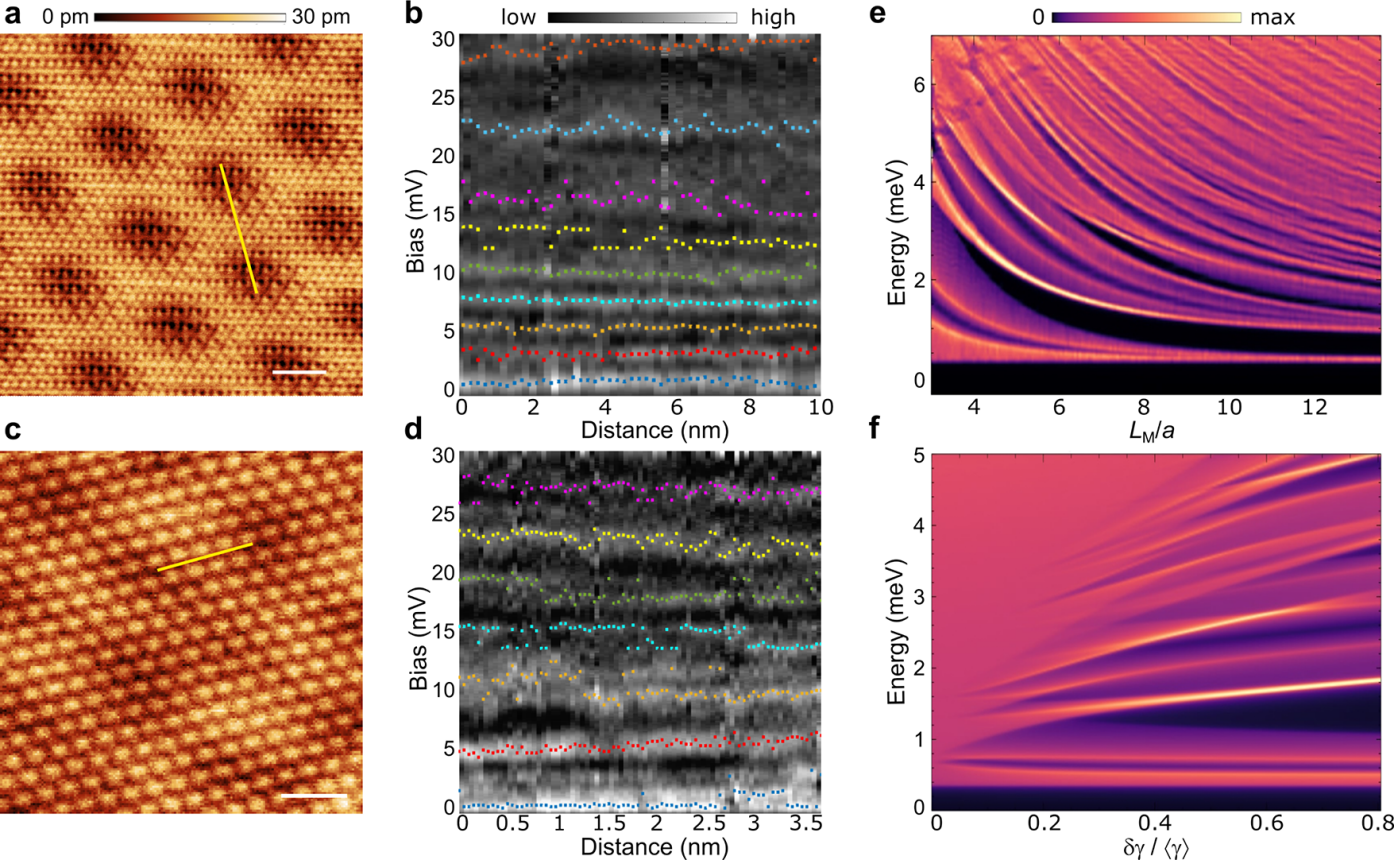
Moiré magnons in CrBr_3_. (a,b) Area with moiré wavelength 7 nm (Scale bar 4 nm. Image bias 1 V). Spatial dependence of antisymmetrized d^2^*I*/d*V*^2^ (b) with spectra taken along yellow line in panel (a). (c,d) Area with moiré wavelength 3.6 nm (Scale bar 2 nm. Image bias 2 V). Spatial dependence of antisymmetrized d^2^*I*/d*V*^2^ (d) with spectra taken along yellow line in panel (c). In (b),(d), colored points indicate the locations of maxima in the d^2^*I*/d*V*^2^ corresponding to the van Hove singularities in the magnon spectral function (see SI). (e,f) Theoretical dependence of the magnon spectral function with the moiré length (e) and with the strength of the exchange modulation (f).

The dependence of low energy inelastic magnon peaks with the moiré wavelengths can be rationalized from the reconstruction of the magnon bands triggered by the moiré pattern. The momentum folding of the magnon structure depends on the length of the moiré pattern, leading to magnon van Hove singularities whose energy location depends on the specific moiré. In particular, longer moiré lengths give rise to magnon van Hove singularities with a smaller energy spacing, as observed experimentally. This phenomenology is captured with the moiré Heisenberg model, as shown in Figure 2e. The relative intensity of the moiré van Hove singularities is controlled by the strength of the moiré modulation as shown in Figure 2f, highlighting that the observation of moiré magnons can allow inferring the value of the real space modulation of the exchange constants. By comparing the theory calculations with the observed spectra we roughly estimate the modulation of the exchange constants of Δ*J*/⟨*J*⟩ ≈ 0.3. This value is consistent with the exchange modulation obtained for twisted van der Waals heterostructures.^39^

While our experiments are consistent with the expectation that the observed inelastic features correspond to magnetic excitations, they could also correspond to inelastic excitations of phonons. However, earlier experiments on tunneling devices have shown that the modes with sufficient electron–phonon coupling are at higher energies (above 25 meV)^47^ than the features we observe in our experiments. Additionally, the magnetic origin of the excitations is usually probed by carrying out experiments under an external magnetic field. We have done these experiments (see SI for the results); however, the HOPG substrate shows very clear and strong signatures of Landau levels at high magnetic fields that completely overwhelm the signal from the magnetic excitations in the CrBr_3_ layer. It is also not possible to subtract the signal from the Landau levels as the Landau level spectra of bare HOPG and HOPG covered by CrBr_3_ are different arising from the sensitivity of the Landau levels to the local potential.^48^ At low-magnetic fields (<0.5 T), the signal due to the magnetic excitations is clearly visible, but the shifts due to the Zeeman energy are too small to be reliably detected.

The presence of moiré magnons can be demonstrated even more convincingly through inelastic quasiparticle interference spectroscopy that allows a direct visualization of the length scale of the moiré magnons. We note that this technique has been used to demonstrate the emergence of quantum spin liquid signatures in monolayer 1T-TaSe_2_.^49^ The differential conductance d*I*/d*V* is proportional to the total number of magnons that can be excited with that energy. In the presence of weak scattering, the total number of magnons will be spatially modulated. The dispersion of the moiré magnons can be directly probed by visualizing the Fourier transform of the spatially resolved d*I*/d*V*, shown in Figure 3a, known as quasiparticle interference (QPI). The signature of moiré magnons is directly visible in the QPI due to the reconstruction of the magnon spectra. Specifically, the Fourier transform of the d*I*/d*V*, in the following denoted as Ξ(ω, **q**) stems from inelastic magnon tunneling processes as Ξ(ω, **q**) ∼ *∫A*(ω, **k**)*A*(ω, **k**+**q**)d^2^**k**, where *A*(ω, **k**) is the magnon spectral function. As a result, the magnon QPI reflects a self-convolution of the magnon dispersion, directly reflecting magnon reconstructions in reciprocal space.

**Figure 3:**
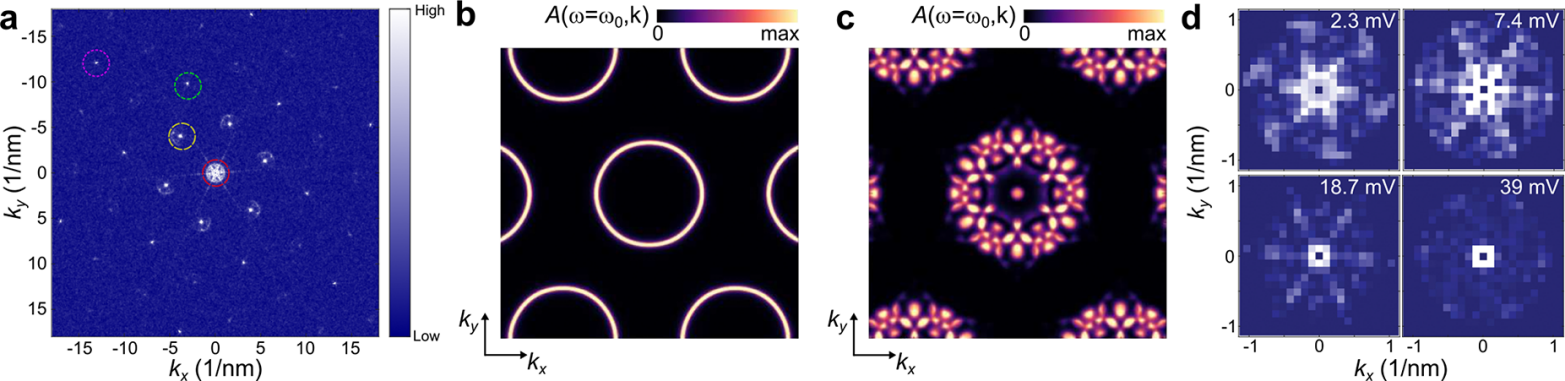
Quasiparticle interference of magnons. (a) FFT of a constant-current d*I*/d*V* map at a bias voltage of 7.4 mV. Red, yellow, green, and magenta dotted circles indicate real space length scales of 7 nm, 1.25 nm, 6 Å, and 4 Å, respectively. (b,c) Calculated momentum-resolved spectral function of magnon *A*(ω, **k**) at constant energy of 5 meV in the absence (b) and presence of the moiré pattern (c). (d) Zoomed-in FFTs of the experimental d*I*/d*V* signal around the Γ-point at the bias voltages indicated in the panels.

To explore the dispersion of the magnonic bands, we performed constant current d*I*/d*V* maps at various energies (parameters mentioned in the SI). The typical FFT of the d*I*/d*V* maps has strong peaks at characteristic reciprocal space points, indicating different topographic periodicities present. The green, magenta, red, and yellow dotted circles in Figure 3a represents Cr–Cr (6 Å), Br–Br (4 Å), moiré (7 nm), and possible  Kekulé distortion (1.25 nm) length scales. The high-symmetry points, especially Γ- and *K-* points have features around them.

Theoretically, in the absence in the moiré pattern, the magnon spectral function at energies below 8 meV should feature a simple circular shape coming from the magnon dispersion ϵ(**k**) ∼ |**k**|^2^ as shown in Figure 3b. This featureless circular shape leads to the well-known disc-like QPI, that does not show a complex angular structure. In stark contrast, in the presence of the moiré, the moiré modulation leads to a full new set features in the magnon dispersion as shown in Figure 3c, as a direct consequence of the magnon moiré mini-bands. The inelastic contribution to the QPI gives rise to the different scattering events associated with the states in Figure 3c, directly reflecting the emergent dispersion of the moiré bands. In particular, the moiré magnon generating QPI will give rise to very short wavelength features appearing around Γ-point in the QPI.

These theoretical moiré QPI predictions can be directly compared with our experimental data. In order to factor out the impact of the topographic moiré modulation in the QPI, we first remove the peaks associated with the moiré length, whose origin is purely structural. Around the Γ-point, after removing the intensity due to the moiré, we see an internal interference pattern strongly dependent on the energy and ultimately vanishing above ∼25 mV (Figure 3d). It must be noted that, in the absence of a moiré pattern, no strong energy dependence of the QPI is expected around the Γ-point. In stark contrast, the presence of moiré magnons leads to an energy-dependent interference pattern around the Γ-point in the full energy window due to the nontrivial interplay between the different magnon moiré bands. The previous phenomenology directly demonstrates the emergence of quasiparticle interference associated with magnons, featuring fluctuations in the moiré length scale and spanning over the whole energy window in which magnonic fluctuations appear in CrBr_3_.

While this kind of QPI features could also arise from elastic scattering between electronic states, it is very unlikely in the present case. First of all, CrBr_3_ is an insulator and has no electronic states close to the Fermi level. We could of course still in principle observe QPI from the electronic states of the HOPG substrate; however, in that case one would expect QPI signal over a large bias range since HOPG has states at all energies. This is in contrast to our experimental results and, hence, the QPI features most likely correspond to the magnon excitations.

To summarize, we have demonstrated the emergence of moiré magnon excitations in 2D monolayer ferromagnet. By using inelastic spectroscopy, we showed that the existence of moiré patterns with different moiré lengths leads to different reconstructions of the moiré spectra. The existence of moiré magnons is further confirmed via inelastic quasiparticle interference, showing the appearance of a dispersion pattern correlated with the moiré length scale. Our results provide a direct visualization in real space of the dispersion of moiré magnons, demonstrating the versatility of moiré patterns in creating emergent many-body excitations.

## Supporting Information Available

Experimental methods (details on the sample growth and STM/STS measurements), determination of the inelastic spectral function, histogramming spatially dependent inelastic excitations, comparison between theoretical and experimental moiré magnon density of states, magnetic field dependence of the inelastic excitations, details on the theoretical model, drift correction and symmetrization of raw images and subtraction of moiré signal at the Γ-point (PDF)
