# Supplementary material for: Visualization of Moiré Magnons in Monolayer Ferromagnet
Source: Nano Lett. 2023 Apr 11;23(8):3412–7. doi: 10.1021/acs.nanolett.3c00417 (PMC10141560; doi:10.1021/acs.nanolett.3c00417)
Supplement: Supplementary file 1 — nl3c00417_si_001.pdf [file nl3c00417_si_001.pdf]

Supporting Information: Visualization of moiré magnons in monolayer ferromagnet

Somesh Chandra Ganguli,*,† Markus Aapro,† Shawulienu Kezilebieke,‡ Mohammad Amini,† Jose L. Lado,*,† and Peter Liljeroth*,†

†*Department of Applied Physics, Aalto University, FI-00076 Aalto, Finland*

‡*Department of Physics, Department of Chemistry and Nanoscience Center, University of Jyväskylä, FI-40014 University of Jyväskylä, Finland*

E-mail: somesh.ganguli@aalto.fi; jose.lado@aalto.fi; peter.liljeroth@aalto.fi

Methods

Sample growth.

The $CrBr_3$ thin film was grown on freshly cleaved HOPG substrates by compound source molecular beam epitaxy. The anhydrous $CrBr_3$ flakes of 99 % purity was evaporated by Knudsen cell. Before growth, the cells were degassed up to the growth temperature 350°C until the vacuum was better than 1×10^{-8} mbar. The growth rate was determined by checking the coverage of the as-grown samples by STM.

STM/STS measurements.

Subsequent to the growth, the sample was transferred to a low-temperature STM (Unisoku USM-1300) housed in the same UHV system. STM imaging and STS experiments were performed at $T = 350$ mK. STM imaging was performed in constant current mode. Differential

conductance (dI/dV) spectra were measured using standard lock-in techniques sweeping the sample bias in an open feedback loop with a.c bias modulation at a frequency of 873.7 Hz. For the dI/dV maps in Fig. 2b,d, the amplitude of bias modulation was 500 μV and the current set point was 500 pA. For constant current dI/dV maps in Fig. 3a,d, the amplitude of bias modulation was kept to 5% of the applied d.c. bias, and the current set point was 200 pA. The raw images were drift corrected by Lawler-Fujita algorithm[S1] and symmetrized to increase the signal to noise ratio of the QPI signal (described in SI).

Determining the inelastic spectral function

The experimental dI/dV signal (Fig. S1a) is numerically differentiated to obtain d^2I/dV^2 (Fig. S1b). The numerical d^2I/dV^2 is smoothened by Savitzky–Golay method to obtain smoothened d^2I/dV^2 (Fig. S1c) and the antisymmetrised d^2I/dV^2 is determined as $(d^2I/dV^2(V)$-$d^2I/dV^2(-V))/2$. The data shown here was a point spectra taken on the 7 nm moiré area.

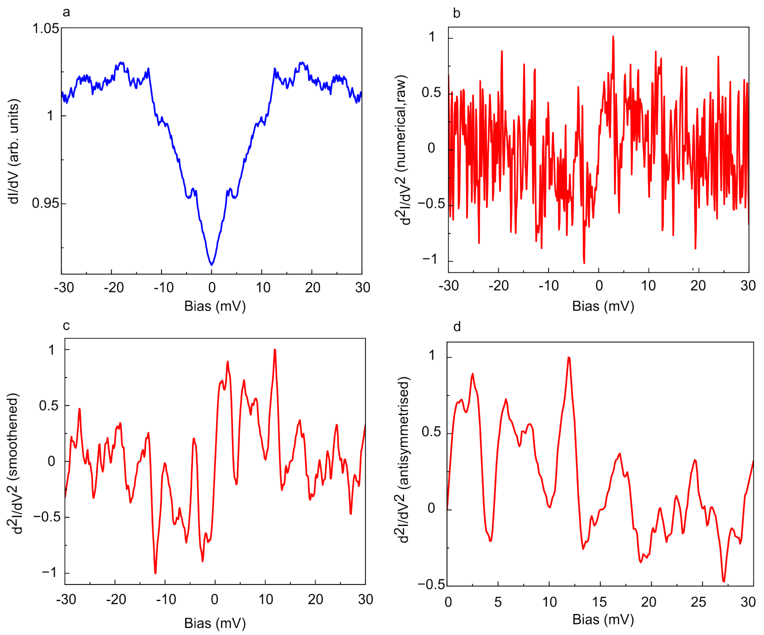

Figure S1: Numerically determined inelastic spectral function: (a) Experimental dI/dV, (b) numerically differentiated d^2I/dV^2, (c) smoothened d^2I/dV^2, and (d) antisymmetrised d^2I/dV^2.

Histogram of spatially dependent inelastic excitations

We determined the energies of the inelastic peaks corresponding to the two different moiré areas in Figs. 2b, d. We observe that, specific number of inelastic peaks appear throughout albeit with some spatial variations in the same moiré area from the histogram plot of the energy values at different spatial locations (Fig. S2a,b). However, the number of inelastic peaks and their energies vary in two different moiré areas, with the larger moiré area having larger number of peaks (Fig. S2a,b).

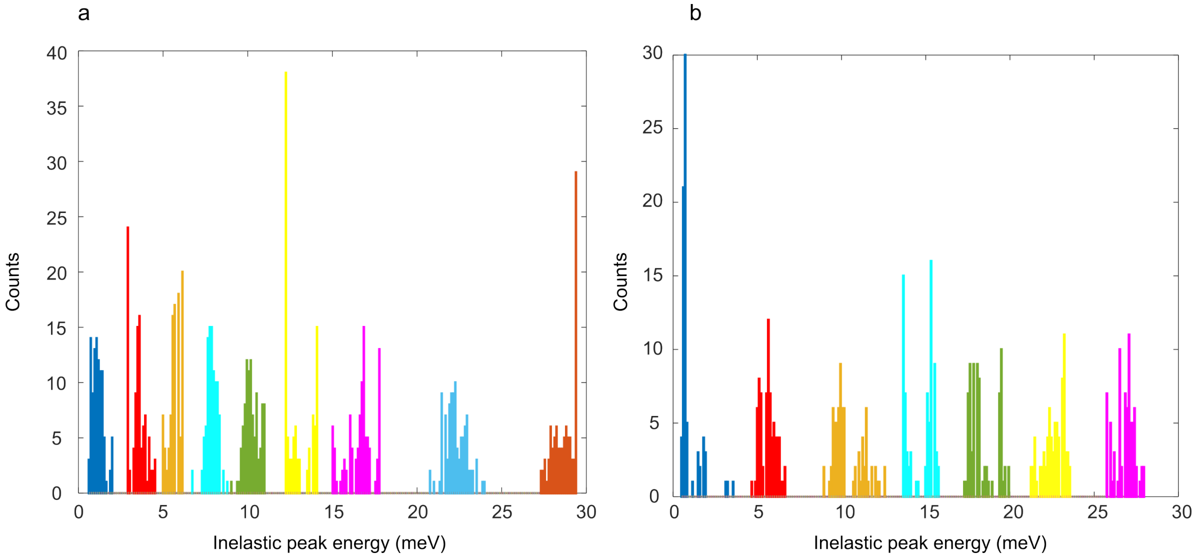

Figure S2: Histogram of inelastic excitation energies corresponding to the (a) 7 nm moiré area, (b) 3.6 nm moiré area. The colors refer to individual miniband energies for the corresponding moiré areas in Figs. 2b, d.

Theoretical and experimental moiré magnon density of states

In Fig. S3a,b, we show the comparison between theoretically calculated density of states (DOS) of moiré magnons and experimentally obtained $\mathrm{d}^2I/\mathrm{d}V^2$ for 3.6 nm and 7 nm moiré areas. It can be noted that the number of experimentally observed peaks in the $\mathrm{d}^2I/\mathrm{d}V^2$ is lower than the number of van Hove singularities in the theoretical DOS. This could possibly

arise from additional energy broadening due to possible disorder in the moiré or short intrinsic magnon lifetime.

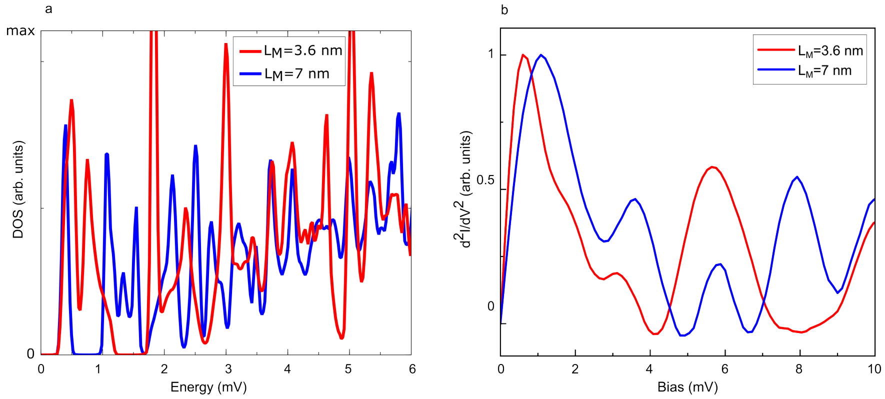

Figure S3: (a) Theoretically calculated density of states (DOS) of moiré magnons and (b) experimental $\mathrm{d}^2I/\mathrm{d}V^2$ for 3.6 nm and 7 nm moiré areas.

Magnetic field dependence of the inelastic excitations

When an external out-of-plane magnetic field is applied, the van Hove singularities in the magnonic band are expected to disperse linearly with the magnitude of the applied field. However, with increasing magnetic field, we observe a change of $\mathrm{d}I/\mathrm{d}V$ signal from having step features to peak-like features (Fig. S4a, b, c). In the presence of an external magnetic field, the substrate HOPG shows strong peak-like features in $\mathrm{d}I/\mathrm{d}V$ (Fig. S4d). They correspond to Landau levels (LL) due to quasi-two dimensional nature of conduction electrons within HOPG.[S2,S3] These strong signals in the $\mathrm{d}I/\mathrm{d}V$ channel from the HOPG increases the number of peak like features observed in $CrBr_3$ and it makes it difficult to quantify the shifts of the magnetic excitations at high magnetic fields.

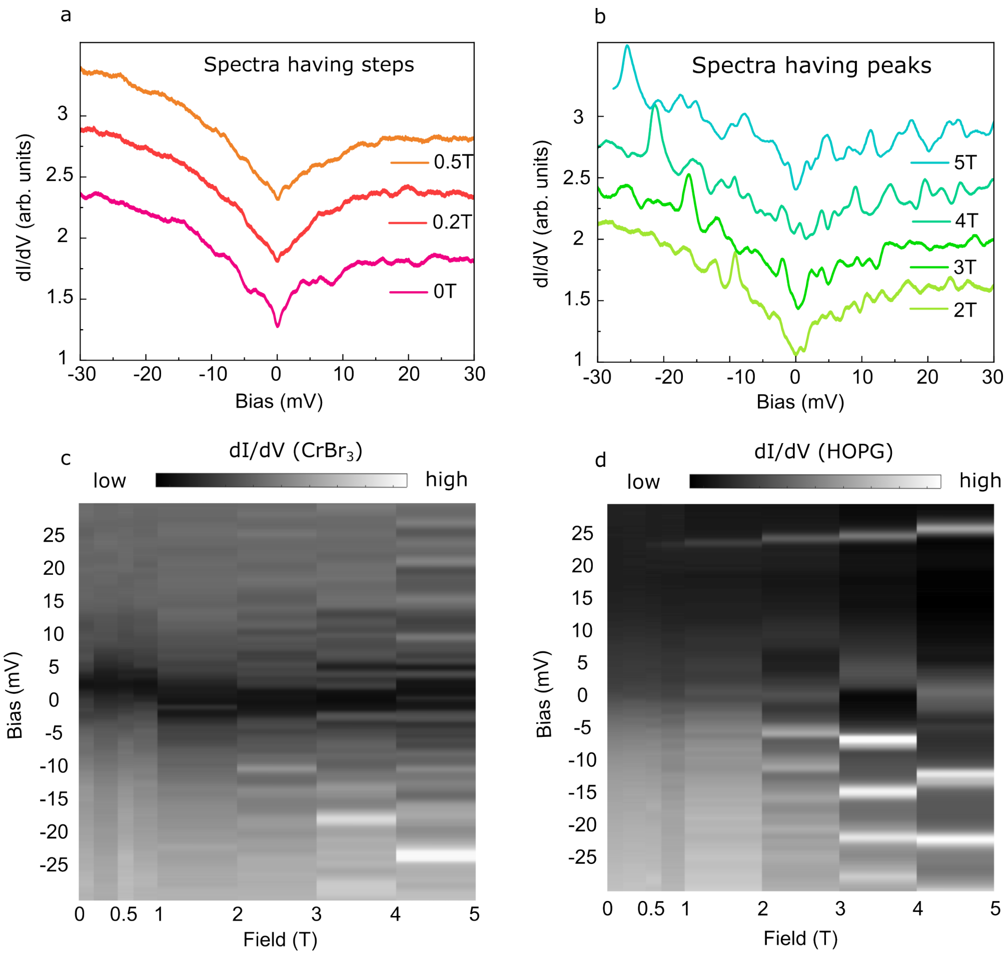

Figure S4: (a, b) Magnetic field dependence of the differential conductance ($\mathrm{d}I/\mathrm{d}V$) on $CrBr_3$ showing step-like features in low magnetic fields (a) and peak-like features in high magnetic fields (b). (c) Full Magnetic field dependence of ($\mathrm{d}I/\mathrm{d}V$) on $CrBr_3$. (d) Magnetic field dependence of the differential conductance ($\mathrm{d}I/\mathrm{d}V$) on HOPG showing the appearance of Landau levels.

Theoretical model

Magnon Hamiltonian

Here we elaborate on the theoretical model accounting for magnetic excitations in $CrBr_3$. The magnetic Cr sites realize a $S = 3/2$ honeycomb lattice, with a Heisenberg Hamiltonian of the form

$$\mathcal{H} = -\sum_{\langle ij \rangle} J_{ij} \vec{S}_i \cdot \vec{S}_j - K \sum_{\langle ij \rangle} S_i^z S_j^z - D \sum_i (S_i^z)^2 + \mathcal{H}_{HO} \tag{1}$$

where J_{ij} are the isotropic exchange coupling, K the anisotropic exchange, D the single ion anisotropic. From the fundamental point of view, the terms K and D give rise to a small gap in the magnon gap, whereas J_{ij} gives rise to the magnon moiré bands. The term $\mathcal{H}_{HO}$ includes additional terms to the Hamiltonian including Kitaev exchange,[S4] biquadratic exchange,[S5] Dzyaloshinskii-Moriya interaction,[S6] and dipolar coupling.[S7] For the sake of concreteness, we will focus our discussion on the modulation of the exchange coupling $J_{ij} = J((\mathbf{r}_i + \mathbf{r}_j)/2)$, which is expected to be the strongest modulation.[S8–S11]

We take as starting point the ferromagnetic state of the Hamiltonian Eq. 1. We derive the magnonic Hamiltonian using a Holstein-Primakoff transformation[S12] in the low temperature regime as $S^z_n = S - a^\dagger_n a_n$, $S^-_n \approx \sqrt{2S} a^\dagger_n$ and $S^+_n \approx \sqrt{2S} a_n$, with $a^\dagger_n$ a_n creation and annihilation magnon operators in site n. The bosonic magnonic Hamiltonian takes the form

$$\mathcal{H} = -\sum_{ij} \gamma_{ij} a^\dagger_i a_j + \Delta \sum_i a^\dagger_i a_i + \text{h.c.} \tag{2}$$

where $\gamma_{ij} \sim J_{ij}$ and Δ controls the magnon gap, leading to a gapless spectra for $D = K = 0$. As a reference, the full magnon spectra of $CrBr_3$ is approximately 25 meV,[S13] consistent with first principles calculations results.[S14]

Moiré magnons

The existence of a moiré pattern leads to a modulation of the exchange constants J_{ij}, in turn leading to a modulation of the magnon hoppings γ_{ij}. From the structural point of view, the spatially dependent stacking leads to a modulation of the super-exchange interaction mediated by the substrate. We show in Fig. S5 the evolution of the magnon spectral function as a function of the moire length L_M of the modulation (Fig. S5a,b) and the strength of the modulation δJ (Fig. S5c,d). Given the underlying structure of the materials, we consider a harmonic profile with C_6 symmetry taking the form

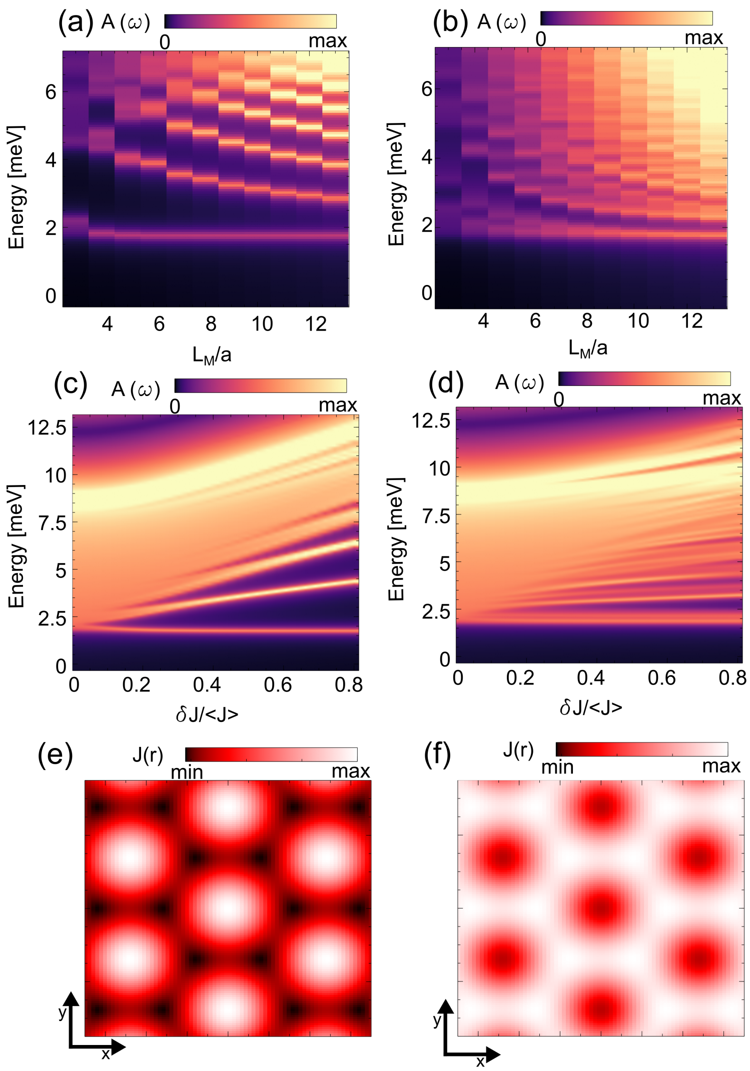

Figure S5: (a,b) Magnon spectral function as a function of the strength of the moiré potential and (b,c) as a function of the moiré length. Panels (a,c) and (b,d) correspond to different exchange modulations, as shown in panels (e,f), respectively.

$$J(\mathbf{r}) = c_0 + c_1 \sum_{\mathbf{G}} \cos \mathbf{G} \cdot \mathbf{r} \tag{3}$$

where $\mathbf{G}$ are the reciprocal lattice vectors of the triangular lattice moire unit cell. We take by definition $\langle J \rangle = \langle J(\mathbf{r}) \rangle$, and $\delta J = \max[J(\mathbf{r})] - \min[J(\mathbf{r})]$, and we consider the two distributions shown in Fig. S5e,f. Panels Fig. S5a,c correspond to the profile Fig. S5e (minimum at $\mathbf{r} = 0$), and panels Fig. S5b,d correspond to the profile Fig. S5f (maximum at $\mathbf{r} = 0$). It is observed that in both cases moiré magnon bands appear at low energies,

and with an energy splitting decreasing as the moiré length gets bigger (Fig. S5a,b). The splitting between low energy modes increases as the δJ modulation becomes stronger, as shown in (Fig. S5c,d).

The appearance of moiré magnons can also be observed in the unfolded magnon structure, as shown in Fig. S6. In particular, the evolution of the magnon spectral function with increasing modulation strength shows the appearance of moiré mini-bands, which are ultimately are responsible of the inelastic quasiparticle interference observed experimentally. This can be observed by computing the isosurfaces of the magnon moiré modes, unfolded to the original moiré unit cell as shown in Fig. S7a,c,e. In particular, it is clearly observed the emergence of short wavelength features in the whole energy range when moiré is switched on. In start contrast, in the absence of a moire pattern (Fig. S7b,d,f) the isosurfaces are featureless. Finally, associated with the magnon reconstruction, the spatial distribution is expected to be affected. In Fig. S8, we show the moiré magnon spectra for a specific moire strength, for

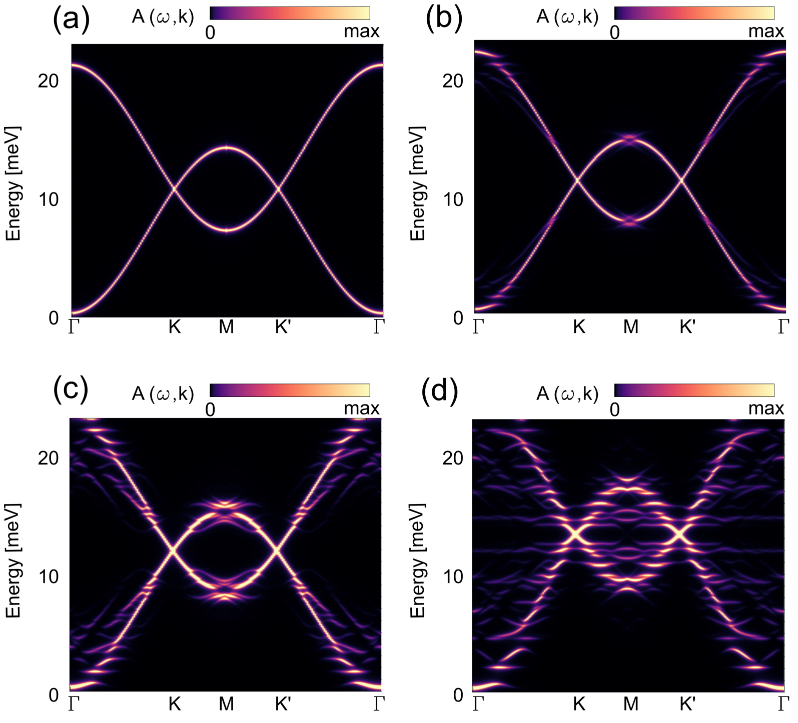

Figure S6: (a-d) Magnon spectral function unfolded to the minimal unit cell, for strength modulation $\delta J = 0$ (a) $\delta J = 0.3\langle J\rangle$ (b), $\delta J = 0.6\langle J\rangle$ (c), $\delta J = 1.2\langle J\rangle$ (d).

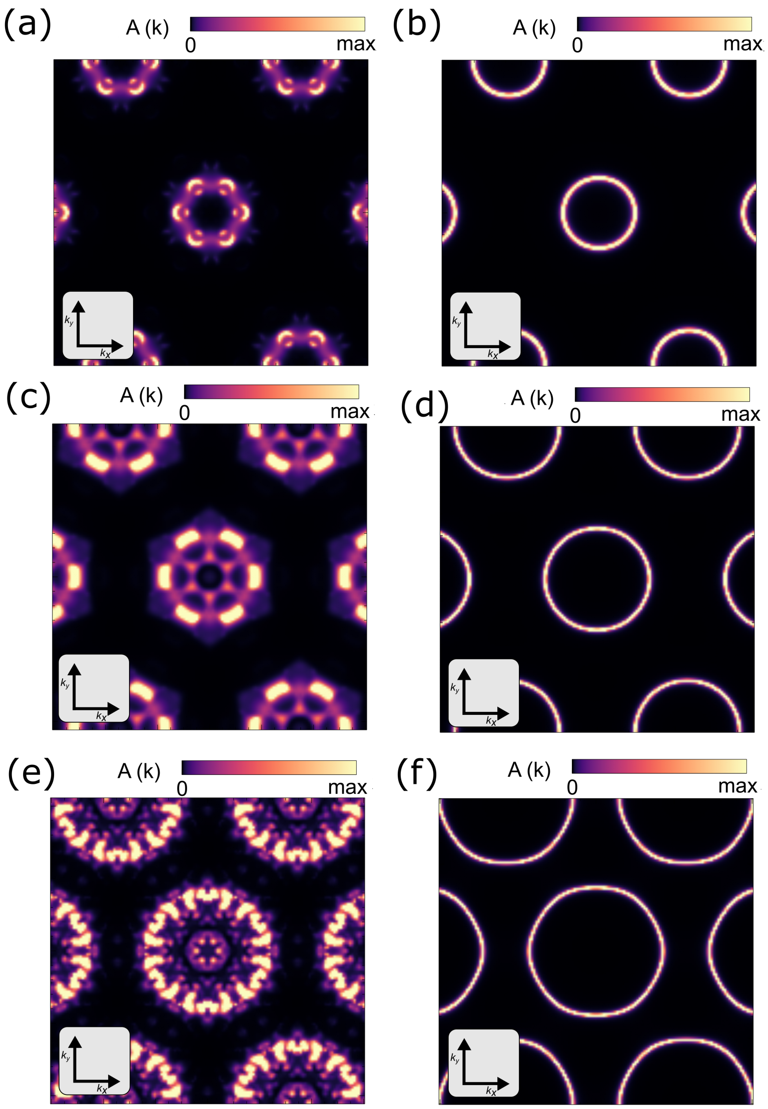

Figure S7: Unfolded magnon spectral function at a fixed energy with (a,c,e) and without (b,d,f) the moiré exchange modulation. In the presence of the moire, the emergence of features close to the Γ point is observed in the whole energy range.

the two profiles shown in Fig. S5e,f. It is observed that the low energy moiré magnons show a non-uniform distribution in the moiré unit cell (Fig. S8c-f), featuring emergent triangular, honeycomb and Kagome magnon lattices.

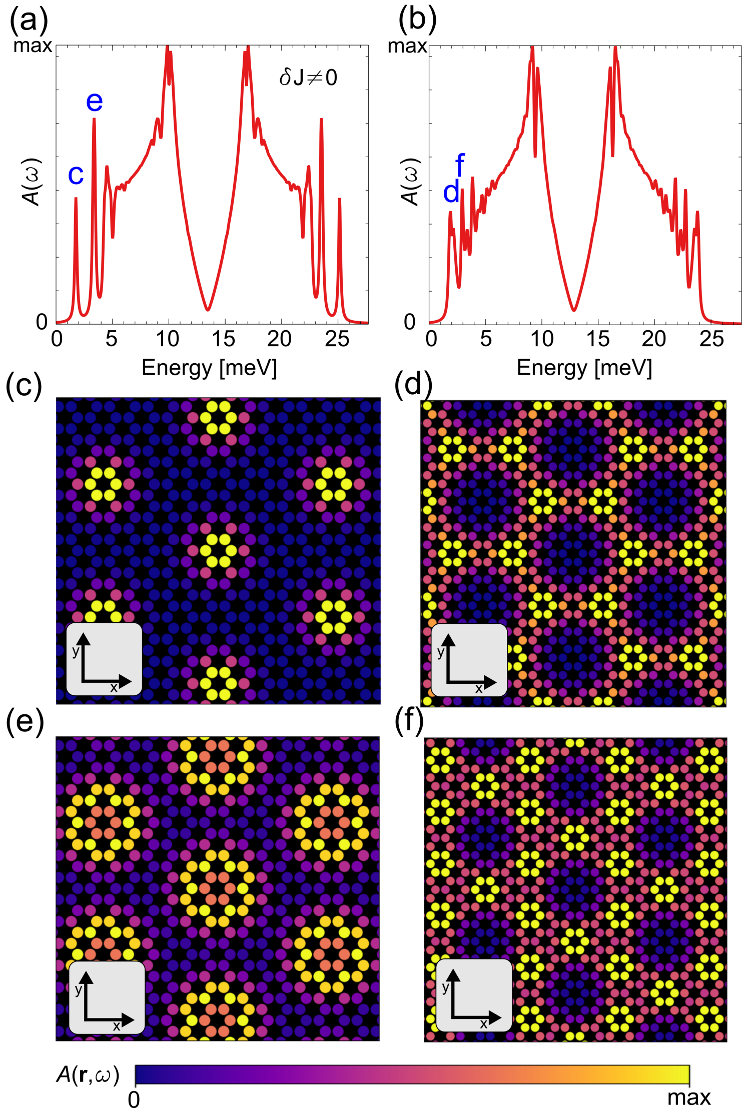

Figure S8: (a,b) Magnon spectral function and (c-f) real-space distribution of the modes marked in panels (a,b). It is observed that the moiré singularities lead to moire modes showing a non-uniform distribution in the moiré unit cell.

Drift correction and symmetrization of raw images

The signal to noise ratio of the raw QPI images are enhanced in two steps. At first, the raw constant current topography and $\mathrm{d}I/\mathrm{d}V$ maps are corrected for both piezoelectric mechanical creep and thermal drift by algorithm developed by Lawler-Fujita.[S1] Finally the drift corrected Fourier transform is symmetrized due to the present C_6 symmetry of the crystal lattice. The raw, drift corrected and symmetrized images are shown in (Fig. S9a-i).

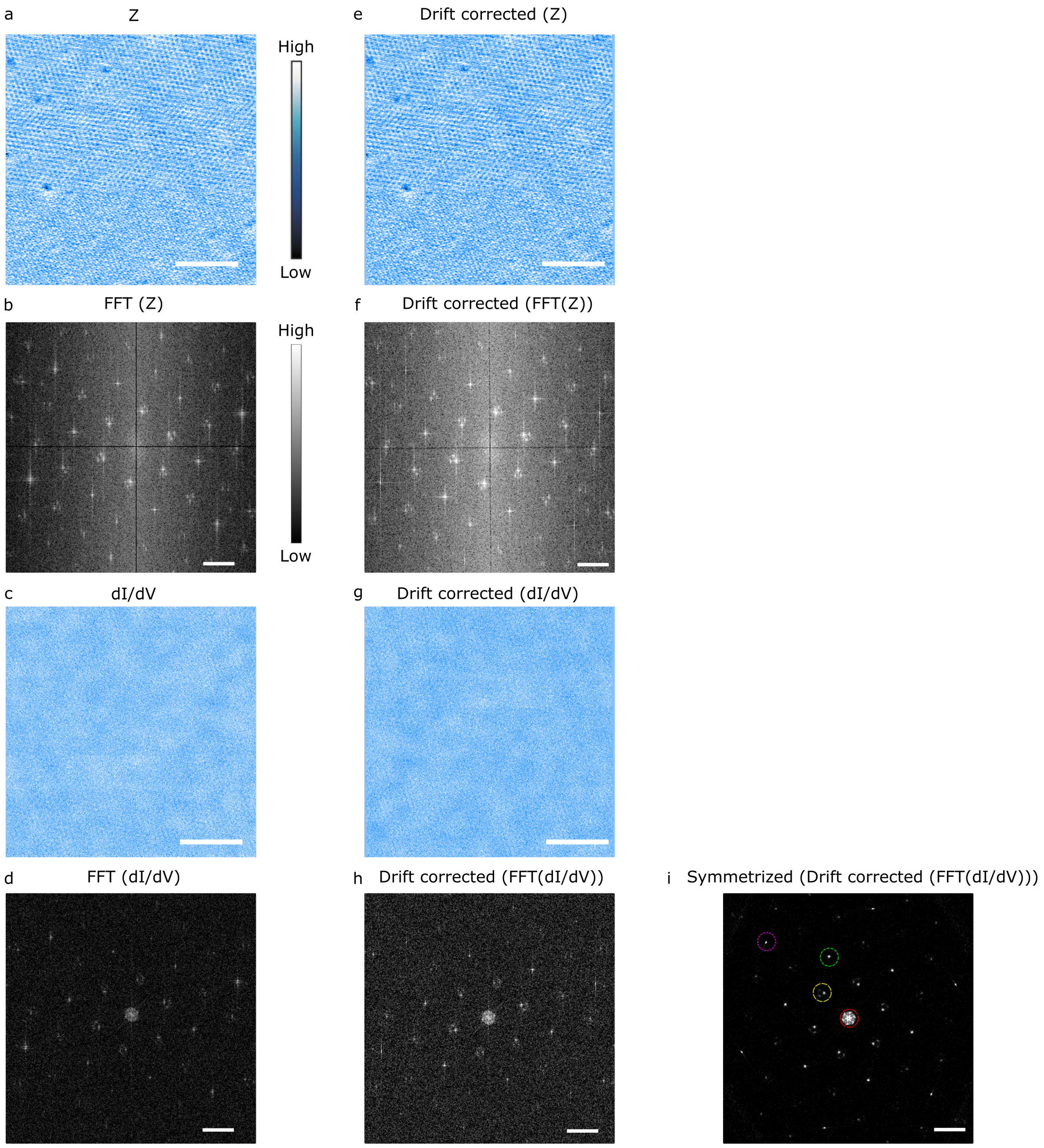

Figure S9: Real space images and fast Fourier transforms (FFTs) after each step: (a,b) Raw topographic image and its FFT, (c,d) raw tunneling conductance ($\mathrm{d}I/\mathrm{d}V$) and its FFT. (e,f) Drift corrected (Lawler-Fujita algorithm) topographic image and its FFT, (g,h) ($\mathrm{d}I/\mathrm{d}V$) and its FFT. (i) 3-fold symmetrised FFT. Constant current map taken at bias V= 2.3 mV, r.m.s modulation=120 μV setpoint= 200 pA. The scale bars for real space images (a,c,e,g) 15 nm and for reciprocal space images (b, d, f, h, i) 5 nm^{-1}. In (i) red, yellow, green and magenta dotted circles indicate real space length scales of 7 nm, 1.25 nm, 6 Å, 4 Å, respectively.

Subtraction of moiré signal at the Γ point

In Fig. S10a, we show drift corrected, 3-fold symmetrised FFT of $(\mathrm{d}I/\mathrm{d}V)$ zoomed in at the Γ point for 7.4 mV. To remove the moiré signal, a circle of uniform intensity was fitted and subtracted around the Γ point. In Fig. S10b, we show the Γ point FFT with moiré signal removed.

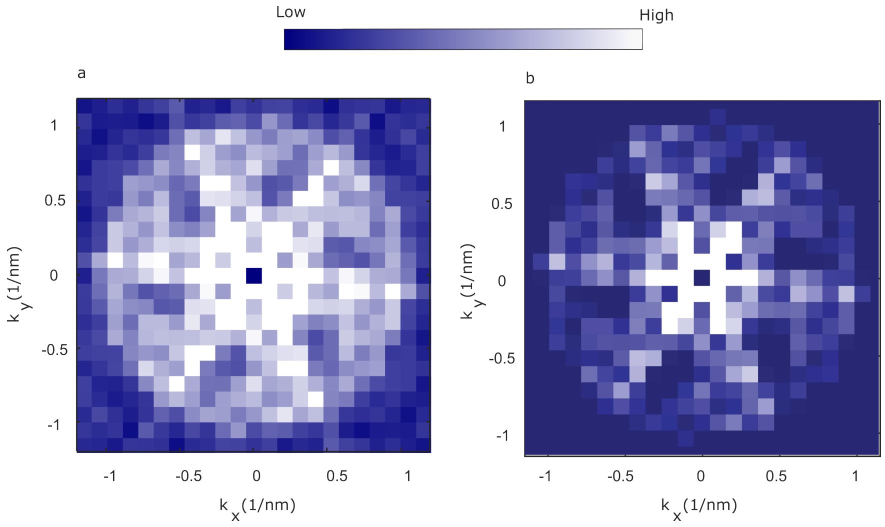

Figure S10: Subtraction of moiré signal from FFT at the Γ point: (a) Drift corrected, 3-fold symmetrised FFT of $(\mathrm{d}I/\mathrm{d}V)$ zoomed in at the Γ point for 7.4 mV. (b) Background-subtracted FFT at the Γ point for 7.4 mV.

References

(S1) Lawler, M.; Fujita, K.; Lee, J.; Schmidt, A.; Kohsaka, Y.; Kim, C. K.; Eisaki, H.; Uchida, S.; Davis, J.; Sethna, J., et al. Intra-unit-cell electronic nematicity of the high-Tc copper-oxide pseudogap states. *Nature* **2010**, *466*, 347–351.

(S2) Li, G.; Andrei, E. Y. Observation of Landau levels of Dirac fermions in graphite. *Nat. Phys.* **2007**, *3*, 623–627.

(S3) Matsui, T.; Kambara, H.; Niimi, Y.; Tagami, K.; Tsukada, M.; Fukuyama, H. STS Observations of Landau Levels at Graphite Surfaces. *Phys. Rev. Lett.* **2005**, *94*, 226403.

(S4) Xu, C.; Feng, J.; Xiang, H.; Bellaiche, L. Interplay between Kitaev interaction and single ion anisotropy in ferromagnetic CrI_3 and $CrGeTe_3$ monolayers. *npj Comput. Mater.* **2018**, *4*, 57.

(S5) Kartsev, A.; Augustin, M.; Evans, R. F. L.; Novoselov, K. S.; Santos, E. J. G. Biquadratic exchange interactions in two-dimensional magnets. *npj Comput. Mater.* **2020**, *6*, 150.

(S6) Chen, L.; Chung, J.-H.; Gao, B.; Chen, T.; Stone, M. B.; Kolesnikov, A. I.; Huang, Q.; Dai, P. Topological Spin Excitations in Honeycomb Ferromagnet CrI_3. *Phys. Rev. X* **2018**, *8*, 041028.

(S7) Lu, X.; Fei, R.; Zhu, L.; Yang, L. Meron-like topological spin defects in monolayer $CrCl_3$. *Nat. Commun.* **2020**, *11*, 4724.

(S8) Soriano, D.; Cardoso, C.; Fernández-Rossier, J. Interplay between interlayer exchange and stacking in CrI_3 bilayers. *Solid State Commun.* **2019**, *299*, 113662.

(S9) Sivadas, N.; Okamoto, S.; Xu, X.; Fennie, C. J.; Xiao, D. Stacking-Dependent Magnetism in Bilayer CrI_3. *Nano Lett.* **2018**, *18*, 7658–7664.

(S10) Chen, W.; Sun, Z.; Wang, Z.; Gu, L.; Xu, X.; Wu, S.; Gao, C. Direct observation of van der Waals stacking–dependent interlayer magnetism. *Science* **2019**, *366*, 983–987.

(S11) Song, T.; Sun, Q.-C.; Anderson, E.; Wang, C.; Qian, J.; Taniguchi, T.; Watanabe, K.; McGuire, M. A.; Stöhr, R.; Xiao, D.; Cao, T.; Wrachtrup, J.; Xu, X. Direct visualization of magnetic domains and moiré magnetism in twisted 2D magnets. *Science* **2021**, *374*, 1140–1144.

(S12) Holstein, T.; Primakoff, H. Field Dependence of the Intrinsic Domain Magnetization of a Ferromagnet. *Phys. Rev.* **1940**, *58*, 1098–1113.

(S13) Ghazaryan, D.; Greenaway, M. T.; Wang, Z.; Guarochico-Moreira, V. H.; Vera-Marun, I. J.; Yin, J.; Liao, Y.; Morozov, S. V.; Kristanovski, O.; Lichtenstein, A. I.; et al., Magnon-assisted tunnelling in van der Waals heterostructures based on $CrBr_3$. *Nat. Electron.* **2018**, *1*, 344–349.

(S14) Zhang, W.-B.; Qu, Q.; Zhu, P.; Lam, C.-H. Robust intrinsic ferromagnetism and half semiconductivity in stable two-dimensional single-layer chromium trihalides. *J. Mater. Chem. C* **2015**, *3*, 12457–12468.
